# Physical Activity intervention effect on wellbeing and overall functioning in an CAMHS low secure service

**DOI:** 10.1192/j.eurpsy.2025.1176

**Published:** 2025-08-26

**Authors:** C. Schneider, E. Stephen

**Affiliations:** 1 RCPsych; 2 BAP, london, United Kingdom

## Abstract

**Introduction:**

Potters Bar Clinic, CAMHS LSU, Elysium Healthcare, collaborates with external physical exercise therapy provider *Psychesoma.* Psychesoma operates within two CAMHS Low Secure wards. Psychesoma works with patients who have mental health problems, ASD, and learning disabilities. Psychesoma offer person-centred group and individual sessions aiming to improve young peoples’ mood state, wellbeing, and health through Physical exercise therapy.

**Objectives:**

We study the influence of physical exercise intervention in positive well-being, psychological distress, and fatigue measures in young people receiving mental health treatment under section 3 of the MHA in a LSU environment.

**Methods:**

The *Subjective Exercise Experience Scale (SEES; McAuley & Courneya, 1994)* is a measure of global psychological responses to exercise stimuli and is a standardised measures used in previous similar research. The SEES assesses three general categories of subjective responses to exercise stimuli: positive well-being, psychological distress, and fatigue. Each sub-scale includes 4 emotion statements, with 12 statements in total. Young people are required to rate the degree to which they feel each emotion in the present moment on a Likert-type scale (1; ‘Not at all’ – 7; ‘Very much so’).

**Results:**

This measure is completed pre- and post- Psychesoma sessions. The data was collected by Psychesoma trainers between December 2022 and November 2023. The measure was completed 63 times in total by 11 young people. On the positive wellbeing sub-scale, the cohort had a mean pre-session score of 11.63, and a mean post-session score of 17.23, representing a 5.31 point increase in positive wellbeing. On the psychological distress sub-scale, the cohort had a mean pre-session score of 9.18 and a mean post-session score of 7.06, representing a 2.21 point decrease in psychological distress. On the fatigue sub-scale, the cohort had a mean pre-session score of 14.73 and a mean post-session score of 10.69, representing a 3.68 point decrease in fatigue

**Conclusions:**

Data indicated that young people experience an increase in positive wellbeing and a decrease in psychological distress and fatigue immediately following Psychesoma sessions. Further research in place to explore the long term duration of these effects.

**Disclosure of Interest:**

None Declared

